# Vulnerabilities to illnesses in women living on the border of the Guiana Shield mines^*^

**DOI:** 10.1590/1980-220X-REEUSP-2023-0010en

**Published:** 2023-09-01

**Authors:** Lise Maria Carvalho Mendes, Nayara Gonçalves Barbosa, Fábio da Costa Carbogim, Daniele Knopp Ribeiro, Ângela Maria e Silva, Ana Karina Bezerra Pinheiro, Flávia Azevedo Gomes-Sponholz

**Affiliations:** 1 Universidade de São Paulo, Programa de Pós-Graduação em Enfermagem em Saúde Pública, Ribeirão Preto, SP, Brasil.; 2 Universidade Federal de Juiz de Fora, Faculdade de Enfermagem, Departamento de Enfermagem Materno-infantil e Saúde Pública, Juiz de Fora, MG, Brasil.; 3 Universidade Federal de Juiz de Fora, Faculdade de Enfermagem, Departamento de Enfermagem Aplicada, Juiz de Fora, MG, Brasil.; 4 Universidade Federal de Juiz de Fora, Programa de Pós-Graduação em Saúde Coletiva, Juiz de Fora, MG, Brasil.; 5 Universidade Federal do Rio de Janeiro, Escola de Enfermagem Anna Nery, Rio de Janeiro, RJ, Brasil.; 6 Universidade Federal do Ceará, Programa de Pós-Graduação em Enfermagem, Fortaleza, CE, Brasil.

**Keywords:** Women’s Health, Mining, Border Health, Health Vulnerability, Vulnerable Populations, Salud de la Mujer, Minería, Salud Fronteriza, Vulnerabilidad en Salud, Poblaciones Vulnerables, Saúde da mulher, Mineração, Saúde na fronteira, Vulnerabilidade em Saúde, Populações vulneráveis

## Abstract

**Objective:**

To analyze the the vulnerabilities to illnesses in women living on the border of the Guiana Shield mines: Brazil, French Guiana, and Suriname.

**Method:**

Descriptive, exploratory field study with a qualitative approach. Data collection took place with 19 women who were living in the mining context, in April 2018. The interviews were recorded and transcribed in full and subsequently analyzed in the light of the concept of vulnerability.

**Results:**

Women aged between 30 and 39 years, predominantly black and brown, on a common-law marriage, multiparous, of low level of education, and with work activities related to mining. Three empirical categories emerged: Exposure to environmental and life conditions in the mines: vulnerabilities to illnesses in women; Sexual and reproductive health in the context of borders: the invisibility between legality and illegality; Gendered facets of violence in the mines on the border of the Guiana Shield.

**Conclusion:**

Vulnerability is marked in the three dimensions of the concept: in the difficult access to health services, in the discontinued treatment, and in the disparity in health policies within countries, which are important aspects of vulnerability and health conditions.

## RESUMO

**Objetivo:**

Analisar as vulnerabilidades para o adoecimento de mulheres em áreas de garimpos da fronteira do Escudo das Guianas: Brasil, Guiana Francesa e Suriname.

**Método:**

Pesquisa de campo, descritiva, exploratória, de abordagem qualitativa. A coleta de dados ocorreu com 19 mulheres que vivenciavam o contexto de garimpagem, em abril de 2018. As entrevistas foram gravadas e transcritas na íntegra e posteriormente analisadas à luz do conceito de vulnerabilidade.

**Resultados:**

Mulheres com idade entre 30 e 39 anos, predominantemente pretas e pardas, união estável, multíparas, baixa escolaridade e com atividades de trabalho relacionadas à garimpagem. Emergiram três categorias empíricas: Exposição às condições ambientais e de vida nos garimpos: vulnerabilidades para o adoecimento de mulheres; Saúde sexual e reprodutiva no contexto de fronteiras: a invisibilidade entre a legalidade e a ilegalidade; Facetas gendradas da violência nos garimpos da fronteira do escudo das Guianas.

**Conclusão:**

A vulnerabilidade é acentuada nas três dimensões do conceito, quais sejam: a dificuldade de acesso aos serviços de saúde, tratamento descontinuado e disparidade nas políticas de saúde entre os países, que são aspectos importantes à vulnerabilidade e condições de saúde.

## RESUMEN

**Objetivo:**

Analizar las vulnerabilidades por la enfermedad de las mujeres en zonas mineras en la frontera del Escudo Guayanés: Brasil, Guayana Francesa y Surinam.

**Método:**

Investigación de campo, descriptiva, exploratoria, con enfoque cualitativo. La recolección de datos se realizó con 19 mujeres que vivían el contexto de las minerías, en abril de 2018. Las entrevistas fueron grabadas y transcritas en su totalidad y posteriormente analizadas a la luz del concepto de vulnerabilidad.

**Resultados:**

Mujeres de 30 a 39 años, predominantemente negras y pardas, unión estable, multíparas, baja escolaridad y con actividades laborales afines a la minería. Surgieron tres categorías empíricas: Exposición a las condiciones ambientales y de vida en las minerías: vulnerabilidades de las mujeres a enfermarse; salud sexual y reproductiva en el contexto de las fronteras: la invisibilidad entre la legalidad y la ilegalidad; facetas de género de la violencia en las minerías de la frontera del escudo guayanés.

**Conclusión:**

La vulnerabilidad se acentúa en las tres dimensiones del concepto, a saber, el difícil acceso a los servicios de salud, la interrupción del tratamiento y la disparidad en las políticas de salud entre países, son aspectos importantes para la vulnerabilidad y las condiciones de salud.

## INTRODUCTION

Brazil and French Guiana are separated by a border region of 730.4 km. The territorial delimitation between Brazil and Suriname goes over 593 km. In these border areas, it is common for people to travel for work, commerce, social activities, and health care. However, these territorial limits have their own cultural aspects, differences in access to social, economic and health care opportunities, which leave individuals in vulnerable conditions^(1,2)^.

Investigations carried out in border areas have to consider aspects such as the dynamics of occupation of these spaces, the composition of populations, mobility, and cross-border interactions^(2)^. In this regard, we will try to situate the nosographic scenario of the Brazilian border with French Guiana and Suriname. This border region includes, on the Brazilian side, the municipality of Oiapoque-Amapá, the military village of Clevelândia do Norte, the National Park of Montanhas do Tumucumaque (*PNMT*), the Uaçá Indigenous Lands (*TI*) and the community of Ilhabela, the latter being a place where clandestine mining activities are carried out, which commonly occur on the foreign side of the border^(3)^. On the Guianese side, there is the twin city of Saint George and the Waiãpi indigenous people living in the commune of Camopi. In this area there are several clandestine mines occupied by Brazilians, among which Sikini stands out. The proximity of the Brazil-Suriname border is a sparsely populated region and there are, in some places, indigenous villages and riverside communities^(3)^.

The inhabitants of this region are predominantly prospectors, small businessmen, and women who carry out ancilary activities associated with gold extraction in the mining environment, such as freight forwarders, vendors, and sex workers^(1–3)^. It appears that these women experience overlapping vulnerabilities; they have little or no education, precarious housing and, often, are hired to work in mining by intimate partners and/or with financial debt^(1)^.

Commuting and disorderly migration, which is intensely carried out, associated with the challenges that the forest imposes for access to health teams, clandestinity, and the fact that these are people invisible to the State, favor the rapid spread of diseases^(4–6)^. Potential health problems include influenza A, malaria, beriberi, digestive disorders, leishmaniasis, dermatitis, worm infestations, syphilis, Chikungunya, Dengue, Covid19, HIV/AIDS, and other infections, as well as snake bites^(1,4–6)^.

Concerning women’s health, current knowledge is extremely limited^(1)^. Women in the mining area constitute a vulnerable group, considering the intersection of gender inequalities, stigma, clandestinity, origin, associated with the geographic isolation of the region. These aspects increase the risk of these women to health problems, such as sexually transmitted infections (STI/HIV), reduced access to rapid testing, difficulties in the sustained use of condoms, difficulties in performing and receiving the screening test for cervical cancer, exposure to sexual violence, exposing weaknesses related to gender and reproductive health in this perspective^(1,5)^.

It was in this context that the concerns and motivations arose for carrying out this study, which proposes to analyze the vulnerabilities to illnesses in women in mining areas on the Guianas Shield border - Brazil, French Guiana, and Suriname.

## METHOD

### Design of Study

Descriptive, exploratory field research, with a qualitative approach, the analysis process of which was based on the concept of Vulnerability^(7)^. This concept emerged in the mid-1990s, in response to the HIV/AIDS epidemic, with the observation that the epidemic responded to determinants that went beyond the pathogenic action of the virus^(8)^. We can summarize it as the model for considering the chance of people being exposed to illness as a result of a set of aspects that are not only individual, but also collective, contextual, and that lead to greater susceptibility to illness, and the likelihood of obtaining resources to protect oneself^(7,8)^.

In compliance with methodological rigor, Equator’s guidelines for qualitative research, set out in the Consolidated Criteria for Reporting Qualitative Research (COREQ), were used.

### Study Local

The study was carried out in the district of Ilhabela-AP, a small island used by prospectors, who carry out clandestine activities in the border region of Brazil with the Guiana Shield, to rest^(1)^. The location was chosen for logistical and safety reasons, as access to mines in France or Suriname is illegal, and is reached through long journeys on foot and/or by canoe, with the dangers inherent to living in the forest.

### Population, Inclusion and Exclusion Criteria, Sampling

The study participants were selected using the snowball technique, by exponential sampling, used to achieve hard-­to-reach groups^(9)^. This way, the participants themselves informed the researcher of other possible women to integrate the study. It should be noted that, due to the high mobility exercised by these women to raid the mines, it was extremely difficult to approach them.

As inclusion criteria, being Brazilian, female, and experiencing the work routine in clandestine mines on the aforementioned border were listed. All invited women agreed to participate in the research. In total, 20 women were included and interviewed. One participant was excluded, due to noises from boats in the audio recording, which made transcription impossible, since, on the day after the transcription, the participant had left to the mines. Data collection ended when the themes brought up by the women during the interviews began to be repeated, demonstrating content saturation^(10)^.

### Data Collection

The immersion in the field lasted for 15 days. The interviews took place from May to June 2018. The period was intentionally chosen by the researcher, as it coincided with the rigorous Amazonian winter and the flooding of the rivers, making boat trips faster and less risky.

The first contact was made with the community leader, who explained to the women that the activities carried out with them would be to investigate their health conditions and reassured them about the issue of clandestinity.

The collection took place through the use of three capacitors to obtain data: 1- non-participant observation, which focused on the social interaction of the participants, highlighting cultural aspects in health modes and practices; 2- record in a field diary, used as an important tool for taking notes during the field immersion period; 3- application of a sociodemographic questionnaire, followed by a semi-structured interview, in a private environment, which took place in the *barrancos* (ravines) - as the women refer to their homes - made of wood and asbestos roofs, on the banks of the Upper Oiapoque River. Data collection techniques were not fixed and rigid. They interspersed with each other, often occurring concurrently, depending on the relationship built between the researchers and the women.

Data were also described daily in the field diary, simultaneously with the first phase of analysis of what was said and observed, and involves postures, gestures, silence, laughter, crying, political, moral and religious values. The writing in the field diary and the reflection on the experience lived also occurred concurrently with the full transcription of the interviews, which allowed the raw material to be reviewed and doubts that arose during transcriptions resolved.

The interviews were carried out by the main author of this article, a nurse, doctoral student in Science and professor at a higher education institution, with experience in conducting and analyzing qualitative research. The study’s slogans were: tell me a little about how you perceive your health, the diseases you always have, and how you deal with it. When you are sick, do you seek treatment? How does this occur? What place do you look for? It should be noted that in some interviews it was necessary to use linguistic resources to better understand the speeches, such as connectives “as”, “when”, “because”.

A pilot study was carried out with a participant to investigate the scope of the slogan. No need for adaptation was detected. Data from the pilot study were part of the study. Some of the interviewees offered little clarifying data, but others turned out to be key informants. The interview lasted from 20 minutes to three hours.

### Data Analysis

With the transcribed material, the analytical-reflective reading began, which synthesized the data, around speeches capable of representing them. It is noteworthy that the categories were constructed using two exposure strategies. The first one was the frequency of occurrence and explanation of vulnerabilities during the speeches; and the second, the specific conditions inherent to the prospecting scenario observed and noted in a field diary by the researcher responsible for data collection. Thus, the nominal categories arranged by the women in this study were structured in: *i) Exposure to environmental and life conditions in the mines: vulnerabilities to illnesses in women; ii) Sexual and reproductive health in the context of borders: the invisibility between legality and illegality; iii) Gendered facets of violence in the mines on the border of the Guiana shield*.

Data presentation took place through the hermeneutic-dialectic method, in which the understanding of the speeches is produced through the consideration of the context in which the individuals are inserted and in the scope of the historical specificity in which it is produced^(11)^. Coding was performed manually by two researchers. A third researcher was consulted when discrepancies were found in the identification of themes.

The analysis of encodings was based on the concept of vulnerability^(7)^. Vulnerability involves the articulated assessment of three interconnected axes: 1. Individual component: level and quality of information that individuals have about a problem, so as to incorporate it into their daily repertoire of concerns, as well as the interest in transforming these concerns into protected and protective practices. It is understood that changing behaviors are not always directly linked to individual will, but also to life contexts and everyday interpersonal relationships. 2. Social component: obtainment of information, capacity of incorporating it into practical changes. It does not depend only on individuals, but on aspects such as access to means of communication, health, housing, education, the possibility of coping with cultural barriers, being free from violent coercion. Social vulnerability therefore reflects the conditions that lead to social well-being. 3. Programmatic component: social resources that individuals need not to be exposed to harm and in a way that these are made available effectively and democratically. It corresponds to the availability and quality of resources, management and monitoring of national, regional, and local programs for prevention and care. However, barriers to accessing care services, inflections in programs, action plans and funding can expose people to programmatic vulnerability^(7)^.

It is noticed that vulnerability proposes to express the potential for falling ill/not falling ill related to the whole and to each of the individuals who share the experience of a certain set of conditions; in this sense, the experience in clandestine mines, located in the Amazon forest, on an international border.

### Ethical Aspects

The research was approved by the Research Ethics Committee of the Universidade Federaldo Amapá, with opinion no. 2.615.138, on April 23, 2018, and complied with resolution 466/2012 of the National Health Council. To maintain the anonymity of the interviewees, an alphanumeric code was adopted in the speeches. Therefore, for the first interviewee, code E1 was adopted, for the second, code E2, and so on.

## RESULTS

As for sociodemographic data, the predominant age group was between 30 and 39 years old (36.84%). with women from the North (57.89%) and Northeast (31.57%) regions. Regarding the color, predominantly self-declared black and brown (68.42%), juxtaposing a profile of low level of education, with illiterate women (31.57%) or women who had not finished elementary school (31.57%). However, one woman declared having finished higher education (5.26%). All participants declared themselves to be cisgender, with a predominance of heterosexual women (94.73%), with sexual activity with a steady partner in the last three months (63.15%). As for marital status, common-law marriages predominated (73.68%), with women with children standing out (89.47%). Regarding rapid testing for STIs, there was a predominance of women who had never taken the test (63.15%). Their occupations in the mining environment were work at home, housemaid, cook, prospector, vendor, gas station attendant, and hairdresser.

The thematic analysis of the speeches allowed the emergence of two empirical categories, already mentioned above and detailed in the course of the following text.

### Exposure to Environmental and Life Conditions in the Mines: Vulnerabilities to Illnesses in Women

A concern present in all interviews was the possibility of being contaminated by methylmercury. Methylmercury is widely used in rudimentary gold mining activities. Study participants are aware of their vulnerable health conditions and the deleterious effects of exposure to methylmercury, but they have few or no strategies at their disposal to stay healthy and protected. This fact reveals precarious living and working conditions that should be mediated by the Brazilian State.

*We get concerned, you know?Because that water there (from the river) is all full of quicksilver (as they call methylmercury). And we drink this water, you know? And make our food. Then we feel weak, I have a lot of weakness, I think it’s because of that, you know? I feel weak all day long. (E8).*

Disease control and treatment is a challenging issue when dealing with populations undergoing commuting. In the areas of clandestine mining, overlapping vulnerabilities are attributed to access to health services, a triangulation between the absence of public power in these sites, added to the situation of clandestinity, which intersect with questions of sex, class and race of those who occupy these spaces; thus, also, on who remains unattended.

Facets of gender inequalities in access to health services can contribute to the worsening of women’s health conditions, such as the lack of a support network to go in search for treatment in distant places where health services are available. Often, women do not find alternatives to seek health services, because they are responsible for the logistics and maintenance of routine and care in the mines, such as cooking, selling, sending gold to third parties who take care of their children in the urban center of Oiapoque, or, even, due to the need to cross extensive geographical paths, added to the difficult journey, conditioned to travel in boats, to weather conditions; all this becomes a major barrier for these women to be assisted by health professionals. Thus, access to health is violated by multiple events, among which the difficulty of getting appointments and medicines in a timely manner, free exams, guidance and health education are highlighted.

*I can’t go to Oiapoque and lose my job as a cook, you know? Because here comes another one and how am I going to support my children who are studying there in Oiapoque? (E9).*

*We go to the city, they tell us to buy the medicine and there isn’t any, they tell us to undergo some tests, there isn’t any, then I don’t have money, right, buddy? To go down this river is two grams of gold and then you still have to go back, you can’t keep going there, especially in the rain (E6).*

Riverside populations in these areas experience endemic neglected infectious and contagious diseases, such as malaria and leishmaniasis, in parallel with an increase in the prevalence of Chronic Noncommunicable Diseases. The different medications made available by the Brazilian and French States become an access barrier for the continuity of treatment of diseases of people living in this border, corroborating the worsening of their health conditions.

*I have high blood pressure, so I got some medicine in São Jorge, then the doctors in Oiapoque didn’t know what it was for, so I couldn’t get any more* (E4). *I already had ‘leish’, but I didn’t do the treatment at the center, no. I got well with my own efforts! (E7).*

Due to the difficulty of accessing health services, self-­medication occurs too much in these places. During the stay in the field, it was also possible to observe that supplies for dressings and other health treatments were sold by the vendors, as well as procedures such as dressings for leishmaniasis, with the cost, in 2018, of six grams of gold (corresponding to approximately R$820.00).

### Sexual and Reproductive Health in the Context of Borders: the Invisibility Between Legality and Illegality

The clandestine situation of women composes with the other situations addressed in the previous category and characterizes the social and programmatic vulnerability of women in the mines.

The precarious structure of health services, lack of professionals and the availability of tests and medications in a timely manner prevent pregnant women from adequately performing prenatal care. The disparities in access to health between the Brazilian and French states are presented in the women’s speeches; access to diagnostic and screening tests show a great difference in supply and availability.

*We don’t do it (prenatal care), I don’t have a doctor here, when we’re there (Suriname), we can’t cross, because they send us (deport) to Belém, Manaus... then we stay right there, in the middle of nowhere, we can’t go down the river and then get there and do not have a consultation. (E1). We have a lot of difficulty, because ... I did an exam there in Cayenna, then the doctor said I had a lump in the uterus and I had to operate, but I came here, then there was no exam in Iapoque [Oiapoque], so I didn’t search for it anymore, if you have to die, die, right buddy? Our day, only God knows! (E18)*.

In this sense, exposure to malarial infections, as well as their relapses, were referenced in all the women’s speeches; by some, with a certain anguish, and by others, as something trivial and commonplace. It appears that malaria during pregnancy is a recurrent event among these women. It is so present that the women themselves are able to identify the type of Plasmodium that has affected them. With the highest risk of obstetric complications, these women seek medical assistance in Macapá, capital of the state of Amapá (AP), located almost 600 ­kilometers from the municipality of Oiapoque, AP, whose route has 100 kilometers of dirt road, conditioned to points of quagmires during the Amazonian winter, which make the roads impassable for vehicles without traction. They are not always able to be assisted in *Saint George* and referred to *Cayenne*, capital of French Guiana, located two hundred kilometers from Oiapoque.

*I had malaria about 20 times, once I had falciparum malaria... it turns out I was pregnant, 7 months pregnant, I had to go to Macapá, I suffered a lot, it was horrible... horrible... I lost the baby, I suffered a lot. (E14). Once I had malaria when I was pregnant, then I was treated in Saint George because my father was from there, they sent me by plane to Cayenna, so I’m almost not here to tell the story, I underwent a miscarriage, I spent more than 40 days in the hospital, I didn’t pay anything, nothing, they even wanted to send me to France. (E12).*

It was observed that the Voluntary Interruption of Pregnancy (VIP) emerges in a very specific scenario, since in French territory it is legalized until the 14th gestational week and in the Brazilian territory, except in cases of anencephalic fetus, risk to the mother’s health, and pregnancy resulting from rape^(12)^ it is considered a crime. Thus, there are legal divergences on the subject of IVP between countries that share this border, motivating Brazilian women to migrate to French Guiana to perform legal abortion or to acquire medications that cause uterine contractions.

Multiparity and the difficulty of providing for children was listed as a motivation for seeking the procedure. These women’s report also mentions the difference in the treatment of health professionals in assisting women who resort to VIP between the two countries. In Brazil, there was little respect and humane care, with reports of violence in the obstetric context and difficult, interrupted or denied access to pharmacological resources that alleviate pain during procedures.

*I already had four children, right, buddy? Then there was no way, ma’am, to feed one more, alone in this world. I had just arrived here. I had to go there [points to the other side of the river, indicating French Guiana] to take it out [interrupt the pregnancy]. There they treated me well, gave me medicine and everything. I had a colleague, she’s here [calls the neighbor], who went to the hospital in Oiapoque and they did everything in cold blood. The poor thing is still in pain and bleeds like a convict. That’s it, right, buddy? It’s either that or seeing the boy go hungry and we don’t want that, right? (E19)*.

### Gendered Facets of Violence in the Border Mines of the Guiana Shield

In addition to violence in the obstetric context, these women face, in their daily lives in the mining, visible and explicit violence, as well as veiled violence that emerges due to the structural context of male hegemony. With greater frequency, physical violence is witnessed in the spaces of clandestine mines; physical, sexual, and psychological violence occurs in the domestic environment, perpetrated by intimate partners, in addition to State violence, via police force, related to the contingency of clandestinity and gold trafficking.

Intimate partnerships with foreigners were considered mechanisms of social ascension and change of status among the mining community. The interviewees usually call a woman or man who obtains French citizenship from a romantic relationship with a person of French origin a “white elephant”.

*There are also the white elephants, who are the ones looking for marriages with the people there (they point to the other side of the river, towards French Guiana), then they all come cocky, thinking they are French. Husbands are all made a fool of (E7)*.

The meaning is the same as the idiomatic expression used colloquially, that is, it characterizes the possession of something that its owner cannot get rid of and whose cost, especially maintenance, is disproportionate to its usefulness or value. Thus, it is observed that people who live on the Brazilian side of this border are subjugated to a hierarchically inferior position, producing a force field of asymmetrical relations between Brazilians and the French. This belief system is disseminated in the collective social imaginary of the community that shares life on both sides of the same border. And, in its turn, it legitimizes physical, symbolic and/or sexual violence, especially against women.

Gender-based violence is enhanced in the mining environment. Domestic violence was associated by women to alcohol consumption by partners.

*He hit me so hard that I couldn’t get up, it was Nega’s husband who helped me, he got there to help, he drinks every day, every single day and then he says I’m no good that I’m a whore, this stuff... but he goes to these clubs here, he goes out every day with a different woman and I have to take it in silence, and there are 10 people here with that disease (HIV)... I ended up here just because of him, I miss my family in Maranhão, sometimes I think about leaving him and come back, but then I don’t have the courage, I know he’s going to come after me and kill me, he really does, I almost died after he hit me... it’s just a question of praying to God. (E18)*

Women describe forms of polygyny among men, and circumscribe the fear that this practice influences the circulation of STI/HIV, which is also portrayed as present on the small island. Some participants reported that their intimate partners have objects inserted between the foreskin and the cavernous body of the penis, called dominoes or *bouglou*. These objects increase the pleasure during the sexual act, but also increase the vulnerability of these women to STIs, corroborating the condition of vulnerability in health by gendered categories.

*There are workers who go to Oiapoque just to put the dominoes, my husband has five. He paid 5 grams of gold on each (...). Women like it so much, because the deal is really good! [laughter]. (E5)*.

Some also report the suffering of living far from friends and family, showing a certain nostalgia for their homeland. Others see mining as a refuge from a life story in which violence was transversal to all stages of life. It was possible to perceive that fear is a daily component in the women’s experience, the fear of being beaten, the fear of being contaminated, the fear of dying, and also the fear of speaking, which can lead to concealment and lack of reporting by women, hindering protection and prevention actions by public policies.

These lives reveal how issues of sex, race and poverty are used as authorization to violence, not only on these women’s bodies, but also on their subjectivities, their possibilities to build their lives. It should be noted that most of the violence experienced by women is made invisible, tolerated and naturalized, especially those orchestrated by the public power. There is an urgent need for these women’s vulnerabilities to be named and discussed, so that avoidable damage to human beings causes perplexity and ceases to be assimilated as ordinary.

## DISCUSSION

The existence of vulnerabilities related to the scenario in which the participants are inserted was observed, such as contamination by methylmercury, clandestinity, poverty, incidence and recurrence of neglected communicable diseases. Gendered vulnerabilities also occur, which are impelled on a daily basis to these women, such as symbolic violence, violence perpetrated by the partner, domestic violence, sexual harassment, verbal harassment, rape and physical aggression. Furthermore, it appears that the women experienced conditions of vulnerabilities that permeate the issue of gender, such as contamination by methylmercury at work, clandestinity, poverty, communicable diseases and their recurrence, thus reaching other issues at the individual, social and programmatic level. These vulnerabilities are inseparable and cannot be analyzed in isolation, they are interconnected and are mutually referred to^(7)^. These conditions were also found in other studies that investigated the context of mining and borders in the same region^(1,2)^.

It was observed, however, that women have knowledge about some aspects that may favor illness, such as the presence of methylmercury in the waters of the Oiapoque River, a source of subsistence. This concern is scientifically justified, since the rivers in this watershed are contaminated and have a high concentration of chromium and methylmercury in fish and water^(13)^. A systematic review has shown that some of the highest levels of human exposure to methylmercury occur in South America, especially among Amazonian riverside populations^(14)^.

Data confirm typical symptoms of Minamata Disease, currently detectable in Amazonian communities and associated with high levels of exposure to methylmercury, such as visual changes^(14)^. The presence of methylmercury in breast lobules of mastectomy samples that contained an invasive carcinoma was detected, suggesting that further studies seek to understand the role of methylmercury in the pathogenesis of breast cancer^(15)^. These data are alarming and demonstrate a clear need for immediate public health intervention in Amazon riverside communities in the face of exposure to methylmercury.

The women’s statements referred to programmatic vulnerability and the urgent need for an analysis of the dimensions of cross-border cooperation in health to allow and facilitate access to health services in these regions. Cooperation agreements, formal or informal, constitute a possible initiative for disease monitoring, detection, and control^(16)^.

In this regard, there is currently a formal agreement between Brazil and France, so that the two countries become jointly responsible for providing assistance in urgent and emergency situations to the populations of these borders. However, there is a contradiction in relation to the official discourse, with stricter rules for cross-border mobility and health care^(17)^.

With regard to health services in the Amazon region, there is a shortage and difficulty in retaining qualified professionals, in addition to the low supply of technologies. The Health teams *Saúde Ribeirinha* and *Saúde Fluvial*, part of the National Primary Care Policy, increased care coverage; however, in the municipality of Oiapoque there is no coverage of these teams^(18)^.

It was observed that the prenatal care provided to the women interviewed was possibly inadequate. Some health surveys found that the North Region of Brazil had the worst scores in the Program for Improving Access and Prenatal Quality^(19)^. The literature points out that there are some obstacles to adherence to prenatal care in remote areas and that they are part of the context of sociocultural aspects experienced in the daily life on the Amazonian border, regarding precarious labor relations, gender violence, linguistic and cultural variability; absence of support networks, in addition to the disorganization of services, which are far away, are slow in terms of the booking system and have restrictions that require financial support by users. These notes may favor the absence of actions and guidelines for preventing malaria relapses, pointed out by the participants^(20)^.

Gestational malaria was observed to recur in this border region. Transmission involves ecological-environmental, biological and social factors that are expressed in the high social vulnerability of the population that circulates in the border zone, favoring the occurrence of outbreaks and the permanence of the disease^(21)^.

Another situation reported by women was multiparity and lack of resources to resort to VIP. In Brazil, between 2006 and 2015, there were 770 maternal deaths, with abortion as the underlying cause. Among the deaths declared as abortion, 1% was related to medical and legal reasons; however, 56.5% were registered as unspecified deaths^(22)^. The profile of women at greater risk of death from abortion were black and indigenous, with low level of education, aged between 14 and over 40, living in the North region and without a partner^(22)^. On the other hand, French Guiana follows the legislation of metropolitan France and VIP can occur up to the 14th gestational week^(23)^.

In recent decades, Brazil has experienced a high drop in fertility. Nevertheless, sociodemographic differences still impact access to reproductive planning in the country^(24)^. The National Health Survey found differences between the type of contraception used within the Brazilian regions. Women residing in the North region are highlighted as the most sterilized and the male condom is the most used contraceptive method, when compared to women from the South and Southeast regions who use oral contraceptives^(25)^.

This fact may be associated with the difficulty that women in the North Region have in receiving supplies for reproductive planning, which involve logistics, distribution, transport and storage issues. The literature shows that this region had the worst levels of availability, remaining below 80% for all supplies evaluated in the three cycles, except male condoms, with a prevalence of 92.4%. The intrauterine device was only present in one third of the Primary Health Units evaluated in the North region and obtained availability in only 11%^(26)^.

Recently, the Ministry of Health in Brazil expanded the scope of nurses’ activities for the insertion, revision, and removal of the IUD to favor access to this highly effective and long-term method, in the quest to guarantee sexual and reproductive rights and promote women’s autonomy^(27)^. It should be noted that this guideline is convenient to promote the reduction of health and social inequalities among women who have very different experiences in a continental and unequal country like Brazil. The precariousness of access to reproductive planning and contraception for unwanted pregnancies denotes individual, social and programmatic vulnerabilities related to the search for VIP by the women participating in this study.

Study carried out in a mining area observed that women who had previous abortions were more likely to suffer sexual violence by non-partners and/or strangers^(28)^. The high immigration flow in mining regions perpetuates a social environment where rape and other forms of sexual violence are prevalent^(28)^.

Alcohol abuse was an aspect associated with physical violence perpetrated by intimate partners in the women’s testimonies. Data from the I National Survey on Alcohol Consumption Patterns in Brazil showed that four out of ten men reported drinking alcohol during an episode of violence against women^(29)^. Thus, physical violence was related to social vulnerabilities linked to the abusive use of alcohol by intimate partners and to programmatic vulnerabilities due to the absence of support and prevention programs for women victims of violence.

Regarding violence against women in the mining environment, it is important to emphasize that these women are in these clandestine spaces compelled by conditions of extreme poverty or because they found in the mining area escapes for situations of abuse and mistreatment experienced in childhood, domestic violence, sexual violence, and violence by marital partners^(1)^. This triggering violence that operated in the transition of women to escape to clandestine mines and constraints imposed on their trajectories and life possibilities are perpetuated or even intensified when these women are faced with the harsh reality of living in the mines.

Understanding the aspects related to women’s vulnerability to violence is an indicator of iniquity and gendered inequalities that go beyond the probabilistic concept of risk. The characteristic of violence also conforms to the cosmovision of a certain community regarding gender roles. To naturalize impelled and structured violence in this category would mean that there are no efficient forms of intervention to resolve it^(30)^.

The present investigation allowed observing that the vulnerability to illness is accentuated and interconnected to the three dimensions related to the concept of Ayres et al.^(7)^. In the individual dimension, restricted access to specialized information on health prevents the incorporation of knowledge, behaviors, and attitudes in everyday actions. In the social dimension, historical gender inequality enhances women’s vulnerabilities to illnesses. In the programmatic dimension, the absence and/or ineffectiveness of public health policies act to reinforce women’s dependence on men and on clandestine mining activities, as well as to marginalize them from access to health services.

As limitations of the study, it is noteworthy that data collection took place at a rest station, located on the Brazilian side of the border, which made it impossible to observe the conditions of vulnerability in health in the mining environments themselves. However, despite these limitations, referring to safety and legality issues, this study allowed giving voice to women who, for a long period, were silenced and placed on the sidelines of public health policies of the State.

## CONCLUSION

The study analyzed the vulnerabilities of women in mines on the border of the Guiana Shield. These women in clandestinity who live in places of difficult access face different forms of individual, social, and programmatic vulnerability. Exacerbated exposure to methylmercury, susceptibility to neglected and infectious diseases, precariousness of health services on the Brazilian side of the border, difficulty in accessing prevention and health promotion services, discontinued treatment and disparity in health policies between the countries that share the border (among them the difference in the dispensation of medicines between countries and the different legislation regarding VIP) were observed. The women also highlighted the inadequacy of access to prenatal care, multiparity and poverty as a motivation for the search for VIP, gendered, symbolic, psychic and physical violence.

Care from Qualified Nursing and multidisciplinary team is essential to increase the coverage and resoluteness of health care for women in the mining region. Health care for women in remote mining areas can be improved through the provision of local or itinerant health services, with the inclusion and strengthening of primary care in these spaces, using places of rest and support as a strategy to reach these populations who exercise commuting. It is essential to offer guidance through health education, individual and collective agency to face vulnerabilities, intersectoral cooperation to resolve the violence perpetrated against these women. Moreover, the need for targeted public health policies and international cooperation partnerships, adapted to the geographic, sociocultural and health situations characteristic of the border region, as well as the presence of the Brazilian State in these spaces, is notorious.
